# Pelvic girdle pain - associations between risk factors in early pregnancy and disability or pain intensity in late pregnancy: a prospective cohort study

**DOI:** 10.1186/1471-2474-11-91

**Published:** 2010-05-13

**Authors:** Hilde Stendal Robinson, Marit B Veierød, Anne Marit Mengshoel, Nina K Vøllestad

**Affiliations:** 1 Department of Nursing and Health Sciences, Institute of Health and Society, University of Oslo, P.O.Box 1153 Blindern, NO- 0318 Oslo, Norway; 2 Department of Biostatistics, Institute of Basic Medical Sciences, University of Oslo, NO-0317 Oslo, Norway

## Abstract

**Background:**

Recent studies have shown high prevalence rates for pelvic girdle pain (PGP) in pregnancy. Some risk factors for developing PGP have been suggested, but the evidence is weak. Furthermore there is almost no data on how findings from clinical examinations are related to subsequent PGP. The main purpose for this study was to study the associations between socio-demographical, psychological and clinical factors measured at inclusion in early pregnancy and disability or pain intensity in gestation week 30.

**Methods:**

This is a prospective cohort study following women from early to late pregnancy. Eligible women were recruited at their first attendance at the maternity care unit. 268 pregnant women answered questionnaires and underwent clinical examinations in early pregnancy and in gestation week 30. We used scores on disability and pain intensity in gestation week 30 as outcome measures to capture the affliction level of PGP. Multiple linear regression analysis was used to study the associations between potential risk factors measured in early pregnancy and disability or pain intensity in gestation week 30.

**Results:**

Self-reported pain locations in the pelvis, positive posterior pelvic pain provocation (P4) test and a sum of pain provocation tests in early pregnancy were significantly associated with disability and pain intensity in gestation week 30 in a multivariable statistic model. In addition, distress was significantly associated with disability. The functional active straight leg raise (ASLR) test, fear avoidance beliefs and the number of pain sites were not significantly associated with either disability or pain intensity.

**Conclusions:**

The results suggest that a clinical examination, including a few tests, performed in early pregnancy may identify women at risk of a more severe PGP late in pregnancy. The identification of clinical risk factors may provide a foundation for development of targeted prevention strategies.

## Background

Pelvic girdle pain (PGP) is common in pregnancy. Recent studies have shown that about 33-50% of pregnant women report PGP before 20 weeks of gestation, and that the prevalence may reach 60-70% in late pregnancy [1-3]. Despite these high prevalence estimates, we have little knowledge about the risk factors for PGP in pregnancy. Previous studies have reported that strenuous work, a pre-pregnancy history of low back pain (LBP), previous PGP, and multipara are associated with PGP in pregnancy [4-9]. Associations between PGP and psychological variables such as catastrophizing, fear-avoidance beliefs and distress have also been reported [8,10]. However, the number of studies are limited and often hampered by either being retrospective or cross-sectional [7,8], or by lack of multivariable analyses in the prospective studies [4-6].

Moreover, the response variables used in previous studies have most often been dichotomous, such as presence of PGP or not, and did not necessarily reflect the severity of the condition. The importance of also using graded scales has recently been pointed out by Croft [11]. In a recent cross-sectional study we used graded scales and showed that women with combined symphysis pain and bilateral posterior pelvic pain in late pregnancy reported more disability than women with fewer pain sites in the pelvis [3]. Others have shown that women with this combination of pain locations were also less likely to recover postpartum than those with more limited pain distribution [12,13].

Clinical management would probably benefit from an early identification of women at risk for developing disabling symptoms later in pregnancy. A number of tests for pain provocation of different tissues and locations in the pelvis are commonly used and recommended [14]. Although both pain provocation tests and functional tests have most often been used for diagnostic purposes [13,15-17], they might also detect processes at an early stage. Previous studies of PGP during and after pregnancy have reported that positive scores on the posterior pelvic pain provocation (P4) test and the functional Active Straight Leg Raise (ASLR) test were associated with disability [3,18,19] and pain [16,18,19]. Furthermore, when blinded assessors were used, relative high frequencies of positive responses to the tests were also reported for pregnant women without pain in the pelvic area [3]. These results could either indicate low specificity or alternatively that the tests could detect subclinical afflictions and thus be valuable in early identification of those at risk for more severe afflictions.

We established a cohort of pregnant women to study the associations between socio-demographical, psychological and clinical factors measured at inclusion in early pregnancy and disability or pain intensity in gestation week 30.

## Methods

This is a prospective cohort study following pregnant women in Norway from early pregnancy to gestation week 30.

### Procedure

**Figure 1:**
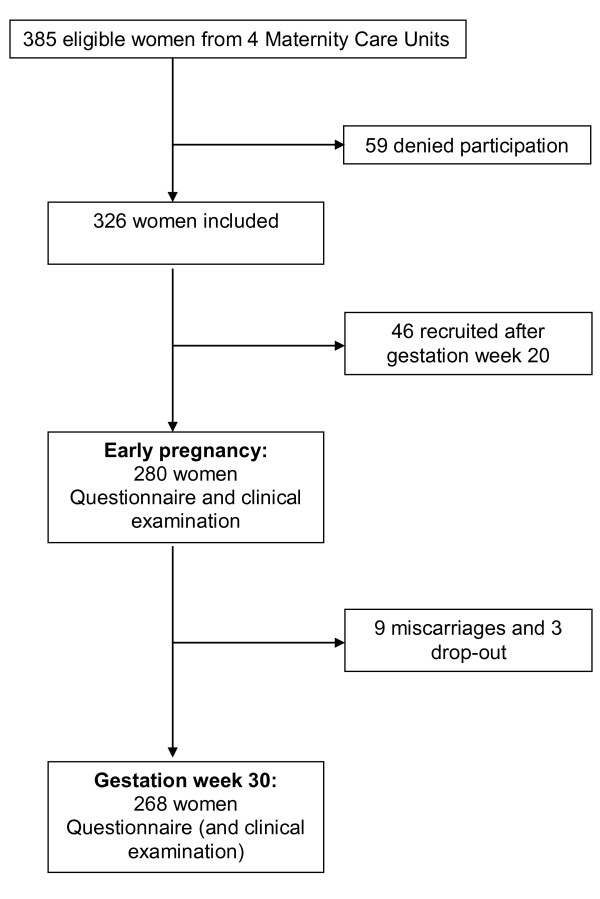
**The study sample**.

The Norwegian public health system offers all women free health services during pregnancy and most women seek special maternity care units (MCUs) for this purpose. We collaborated with four public MCUs in this study, one was located in central Oslo (capital, about 580 000 inhabitants), and the other three covered one entire community (about 24 000 inhabitants) just outside Oslo. Eligible participants were Norwegian-speaking women, who registered at these four MCUs between January 2006 and June 2007. Women not expected to have a normal pregnancy (as determined by the midwives) were excluded. Out of 385 eligible women, 326 gave their informed consent for participation. Out of these 326 women, 280 were included before they reached 20 weeks of gestation, and were thus defined as being in early pregnancy (figure 1). From the time of inclusion to gestation week 30, there were 3 drop-outs and 9 miscarriages among the 280 women included early, thus 268 women participated in gestation week 30 and these constituted our study sample.

After inclusion all answered a comprehensive questionnaire assessing sociodemographic variables, pain locations, pain intensity and disability, distress, and fear-avoidance beliefs. The questionnaire also included questions on general health, health-related quality of life, health locus of control, use of contraceptives, other complaints, and physical activities, variables that were not used in this part of the study. The registered gestation week refers to the week the women were included to the study and completed the questionnaire.

All women were clinically examined in early pregnancy by one of two physiotherapists with post-gradual education in manual therapy. This examination was performed as closely to the inclusion date as possible. Mean time difference between answering the questionnaire and being examined was 1.1 week (SD 1.7 weeks). The clinical examination included six pain provocation tests for the pelvic joints as well as the functional ASLR test and Beighton score for hyper mobility. Other clinical tests were also included, but were not used in this part of the study. The examiner was blinded for all questionnaire data. In gestation week 30, the women filled in a new questionnaire assessing the same elements as at inclusion and underwent a corresponding clinical examination. Data from the clinical examination in gestation week 30 was not used in this part of the study. The Regional Committee for Medical Research Ethics and the Norwegian Social Science Data Services gave formal approval for the study.

### Measurements of response variables

Disability and pain intensity were obtained from questionnaire data collected in gestation week 30. Disability was measured by the Disability Rating Index (DRI), consisting of twelve visual analogue scales (VAS) measuring the ability to perform activities of daily living [20]. The scales ranged from 0 - 100 mm, where the end points were "ability to perform activity without restriction" and "inability to perform the activity", respectively. The twelve activities were: dressing without help, outdoor walks, climbing stairs, sitting for a longer time, standing bent over a sink, carrying a bag, making a bed, running, do light work, do heavy work, lifting heavy objects, participating in exercise/sport. DRI was calculated as the mean of the twelve scales. In order to allow the assessment of disability in women with and without PGP, we chose DRI because it measures disability by limitations in daily activities independent of pain. DRI has previously been applied in studies of pregnant women [2,10], and we also evaluated the items to be adequate for this group.

Pain intensity was measured by the response to the following question: "How strong is your worst evening pain before going to bed?" Since PGP has been suggested to increase with activity [9,17], we chose the intensity of the worst evening pain as the most relevant measure for their experienced degree of pain affliction. The response was measured by a 0-100 mm VAS and the end points were "no pain" and "unbearable pain".

### Measurements at inclusion in early pregnancy

Potential risk factors for PGP were measured by questionnaire and clinical examination at inclusion in early pregnancy.

#### Questionnaire data

Socio-demographical data included age (years), parity (0, 1, ≥ 2 children), marital status (single, married/cohabitant), education (≤ 12 years of school attendance, ≤ 4 years at university, > 4 years at university), use of contraceptive pills last year before pregnancy (yes, no), smoking (yes, no), physical activity before pregnancy (none, < 2, 2 - 4, > 4 hours per week), full time work (yes, no). Pre-pregnancy body mass index (BMI, weight/height^2^) was calculated from self-reported height and weight.

The working condition was identified from the question: "How would you describe your work situation?" With four response alternatives: 1) Most of the time seated; 2) I have to walk a lot; 3) I walk and lift objects; 4) Heavy work. PGP was assumed to increase with weight bearing activities like walking and lifting objects [6,9]. Working condition was categorized as mostly seated work (response alternative 1) and heavy work (response alternatives 2-4).

The Hopkins Symptom Check List (HCSL-25) was used to measure distress (self-reported symptoms of anxiety, depression and somatisation) [21]. Twenty-five symptoms were recorded on a scale from 1 (not bothered) to 4 (extremely bothered). The average value was calculated to obtain the HSCL-25 score. We used a cut off value of 1.75 as established for women by Sandanger and co-workers (1998), and the cut-off reflected non-specific distress, rather than a psychiatric diagnosis [22].

Fear avoidance beliefs was measured by the modified Fear Avoidance Beliefs Questionnaire (mFABQ) [23]. This includes four of the items from the part concerning activity in the original Fear Avoidance Beliefs Questionnaire [23,24]. We chose the modified version because it was possible to answer also by individuals without pain. In accordance with the work from Linton and co-workers [23], the following instructions were given in the questionnaire: "Some women are likely to be afflicted by pain in the back and pelvis during pregnancy. For research purposes, we would like to know if you believe that there is a relationship between such afflictions and activities. Please circle the number on the scale that best corresponds to your belief for each of the following statements". The scale ranged from 0 (total disagreement), to 6 (total agreement), and the total score on mFABQ ranged from 0-24 [23].

Pain locations within the pelvic area (PGP) were determined by a pain drawing filled in by the women before the clinical examination. After the examination, the women were asked to point out the pain sites on their body and, if necessary, the examiner corrected the pain drawing to reflect the areas pointed out. The pain locations in the pelvic area were subsequently coded: no PGP, pain in symphysis only, only posterior pain (uni- or bilateral), combined symphysis pain and unilateral posterior pain, and combined symphysis pain and bilateral posterior pain [3,25]. The two latter categories were collapsed in the analyses (combined symphysis and posterior pain) because of low frequencies.

The number of pain sites was calculated from the questions where the women were asked if they have pain (yes, no) in the neck, shoulder and arms, between the shoulder blades, in the knees. The sum score (0-4) was used in the analyses. Pain located in the area of the lower back and the pelvis was not included in this sum.

Pre-pregnancy history of LBP was identified from the question: "Have you suffered from LBP before you were pregnant (yes, no)?"

#### Clinical examination

Beighton score was used as a measure for joint laxity and consists of 9 tests [26,27]: hyperextension of the knees (yes, no), hyperextension (>10°) of the elbows (yes, no), passive apposition of the thumbs to the flexor aspect of the forearm with straight elbow (yes, no), passive hyperextension of the 5th metacarpophalangeal joints ≥ 90° (yes, no), forward flexion of the trunk, with knees straight, so that the palms of the hands rest easily on the floor (yes, no). The angles were measured with a goniometer. A sum score (0-9) was made of the results of all the tests and hypermobility was defined as a sum score of four and above [26].

We used one functional test, the ASLR test, and six pain provocation tests: the P4 test, the distraction test, the compression test, the Patrick Faber test, the palpation test of the symphysis pubis and the long dorsal sacroiliac ligament (LDL). All the tests have been commonly used and have shown moderate to excellent inter-rater reliability [17,18,28,29].

The active straight leg (ASLR) test [18]: The ASLR test was performed with the women in a supine position with straight legs and feet about 20 cm apart. The women lifted each leg separately about 20 cm above the couch. She was asked to score the difficulty on a six-point scale from 0 (not difficult to lift) to 5 (impossible to lift). The scores on both sides were added and the total score ranged from 0-10. In accordance with previous studies, we considered an ASLR sum score of 4 and above as a positive test [30,31].

The Posterior Pelvic Pain Provocation (P4) test [17]: The P4 test was performed with the women in a supine position. The hip and knee on the tested side were flexed to 90°. The examiner stabilized the contra lateral side of the pelvis while a graded force was applied on the flexed knee into the pelvis along the longitudinal axis of femur. Adduction of the hip was avoided. It was recorded whether a familiar pain was felt in the posterior part of the pelvis on the provoked side (yes, no). Both left and right side were tested and scored separately.

Distraction test: The women were examined in supine position. The examiner applied cross-armed pressure to the anterior superior iliac spines (ASIS) directed laterally. This procedure was assumed to stretch the anterior sacroiliac joint ligaments and to give compression in the dorsal part of the sacroiliac joints. The pain response was recorded (yes, no).

Compression test: The woman were examined in side lying position, knees and hips slightly flexed. Pressure was applied vertically into the pelvis when the examiner leaned her chest against the uppermost iliac crest. The test was assumed to stretch the posterior sacroiliac joint ligaments and compress the anterior part of the sacroiliac joints. The pain response was recorded (yes, no). Both sides were tested and scored separately.

Patrick-Faber test: The women were examined in supine position. The examiner led the ipsilateral leg into flexion, abduction and external rotation so that the heel rested on the opposite kneecap. The examiner stabilized the contralateral side of the pelvis to ensure that the lower back stayed in a neutral position. The ipsilateral knee was lowered against the table and the examiner applied a light overpressure to the subject's knee. It was assumed that both the anterior sacroiliac ligament and the hip joint were stressed [32,33]. The pain response was recorded (yes, no). Both sides were tested and scored separately.

Palpation of the pubic symphysis: The women were examined in supine position. The examiner applied gentle pressure to the pubic symphysis with her hand (flat fingers). If the pressure caused pain that persisted more than 5 seconds after removal of the hand, it was recorded as pain (yes, no).

Palpation of the long dorsal sacroiliac ligament test: The women were examined in side lying position and the examiner palpated the long dorsal sacroiliac ligament at her uppermost side, caudal of the posterior-superior iliac spine. The test was positive if the palpation provoked pain and recorded (yes, no). Both sides were examined and scored separately.

Apart from the P4 test, a sum score was calculated from numbers of positive responses to pain of all the above described pain provocation tests, ranging from 0 (all negative) to 8 (all positive). We decided to use the responses on the P4 test as a single response and not as part of a sum; based on the tests relevance for PGP reported in previous studies [17,34].

### Statistics

Descriptive data are given as frequencies, percentages, means and standard deviations (SDs) or medians and ranges. Multiple linear regression analysis was used to study the associations between potential risk factors measured in early pregnancy and DRI or pain intensity in gestation week 30. Associations between the explanatory variables as well as between the explanatory variables and each of the response variables were studied by Pearson correlation coefficients. The explanatory variables showing significant relationship with the response variable were entered into a multiple regression model. The best subsets of explanatory variables were selected through exclusion of the variables with the smallest contribution to the model (the largest p-values). Two adjusted models are presented for each of the response variables, without (model 1) and with (model 2) adjustment for DRI or pain intensity at inclusion in early pregnancy. The residuals were examined to check model assumptions. The statistical analyses were conducted in SPSS version 16.0 and a 5% level of significance was used.

A continuous variable was the main outcome in the power calculations. The level of significance was set to 5% (two-sided) and the power 80%. Assuming a correlation of medium size, 0.3, in the population, a sample size of 85 is required for assessing significance of a correlation coefficient in the sample [35]. In a multiple regression analysis with five independent variables, the required sample size is 91 to detect a medium effect size of 0.15 (R^2^/(1-R^2^)) [35].

## Results

Mean gestation week at inclusion in early pregnancy was 14 weeks (SD 3 weeks) for the 268 women participating in this study. They were 18 to 45 years old and 59% were pregnant with their first child. Characteristics of the participants are presented in table 1. A total of 59 women declined participation in the cohort study. There were no difference between participants and non participants with regard to age (mean 31 years and SD 4 years in both groups) and marital status. The non-participants (n = 59) were asked about participation in mean gestational week 15 (SD 6 weeks), and 44% were nulliparous. The women excluded from analyses (n = 46) due to inclusion later than gestation week 20 were a little older (mean age 32 years, SD 4) and 77% were nulliparous.

**Table 1:** Characteristics of the women at inclusion in early pregnancy (n = 268)

		Frequency (%)	Mean (SD)
Age (years)			31 (4)
Parity	0	157 (59)	
	1	86 (32)	
	≥ 2	25 (9)	
Gestation week			14 (3)
Marital status (single)		7 (3)	
Education	≤ 12 years school attendance	46 (17)	
	≤ 4 years university	113 (42)	
	>4 years university	109 (41)	
Contraceptive pills, year before pregnancy (yes)		103 (38)	
Pre-pregnancy BMI (kg/m^2^)			23.3 (3.5)
Smoking (yes)		11 (4)	
Physical activity before pregnancy	None	11 (4)	
	< 2 hours per week	84 (31)	
	2 - 4 hours per week	138 (52)	
	> 4 hours per week	34 (13)	
Full time worker (yes)		228 (85)	
Heavy work (yes)		96 (36)	
mFABQ (0-24)			9.3 (3.8)
HSCL-25 (score ≥ 1.75)		38 (14)	
Pre-pregnancy history of LBP (yes)		131 (49)	

BMI, Body Mass Index; mFABQ, modified Fear Avoidance Beliefs questionnaire; HSCL-25, Hopkins Symptom Check List; LBP, Low Back Pain

Fifty percent of the participants reported pain in the pelvic area in early pregnancy and most of them reported posterior pain only (39%) (table 2). Pain in the symphysis only and combined symphysis and posterior pain were reported by 4% and 7% of the women, respectively. The frequencies of negative responses were high on all the clinical tests (54 - 94%). The sum of pain provocation tests had a median value of 1 (range 0, 6) (table 2). Both DRI and pain intensity increased from early pregnancy to gestation week 30, and showed large variation among the women (table 2).

**Table 2:** Distribution of possible risk factors and outcome variables. (n = 268)

		Frequency (%)	Median (range)
Beighton score	Normal (sum<4)	46 (17)	
	Hypermobile (sum ≥ 4)	222 (83)	
Pain locations	No pain	135 (50)	
	Pain in symphysis only	11 (4)	
	Posterior pain only	105 (39)	
	Combined symphysis and posterior pain	17 (7)	
P4 test	Negative	161 (60)	
	Unilateral positive	53 (20)	
	Bilateral positive	54 (20)	
ASLR test	sum<4	240 (90)	
	sum ≥ 4	28 (10)	
Distraction test	Negative	207 (77)	
	Positive	61 (23)	
Compression test	Negative	251 (94)	
	Unilateral positive	15 (5)	
	Bilateral positive	2 (1)	
Patrick-Faber test	Negative	191 (72)	
	Unilateral positive	39 (14)	
	Bilateral positive	39 (14)	
Palpation of pubic symphysis	Negative	241 (90)	
	Positive	27 (10)	
Palpation of LDL	Negative	145 (54)	
	Unilateral positive	41 (15)	
	Bilateral positive	79 (30)	
Sum of pain provocation tests			1.0 (0,6)
DRI in early pregnancy			13 (0,93)
DRI in gestation week 30			36 (0,81)
Pain intensity in early pregnancy (worst evening pain)			0 (0,82)
Pain intensity in gestation week 30 (worst evening pain)			14 (0,99)

P4 test, Posterior Pelvic Pain Provocation test; ASLR test, Active Straight Leg Raise test; LDL, Long Dorsal Sacroiliac Ligament

The correlation coefficients between the potential risk factors and DRI ranged from -0.07 to 0.54 and between potential risk factors and pain intensity ranged from -0.10 to 0.46 (table 3). The correlation coefficients between the potential risk factors ranged from -0.25 to 0.56 and did not suggest collinearity (data not shown). Pain intensity and DRI in gestation week 30 were significantly correlated (r = 0.63, p < 0.001) (table 3).

**Table 3:** Correlation between outcome variables and possible predictors measured at inclusion in early pregnancy (n = 268)

	DRI gestation week 30	Pain intensity gestation week 30
Pain intensity gestation week 30 (worst evening pain, VAS)	0.63***	
Age (years)	-0.07	-0.10
Parity (0, 1, 2 or more)	0.15*	0.18**
Gestation week in early pregnancy	0.03	-0.04
Civil status (married, cohabitant; yes, no)	0.14*	0.22***
Education (≤ 12 years of school attendance, ≤ 4 years university, >4 years university)	0.19**	0.17**
Contraceptive pills, year before pregnancy (yes, no)	-0.13*	-0.04
Pre-pregnancy BMI (kg/m^2^)	0.09	0.12*
Smoking (yes, no)	0.06	0.12
Physical activity before pregnancy (none, <2, 2-4, ≥ 4 hours per week)	-0.05	-0.001
Full time worker (yes, no)	0.05	0.09
Work condition (mostly seated/heavy work)	0.03	0.06
Beighton score for hypermobility	0.01	-0.06
Pain locations (no pain, symphysis pain, posterior pain, combined symphysis and posterior pain)	0.36***	0.44***
P4 test (bilateral negative, uni-/bilateral positive)	0.41***	0.39***
Sum of pain provocation tests (0-8)	0.40***	0.40***
ASLR test (<4, ≥ 4)	0.18**	0.11
HSCL-25 (<1.75, ≥ 1.75)	0.26***	0.15*
DRI in early pregnancy (0-100)	0.54***	0.34***
Pain intensity in early pregnancy (worst evening pain, VAS)	0.44***	0.46***
Pre-pregnancy LBP (yes/no)	0.09	0.10
Number of pain sites (0-4)	0.19**	0.14*
mFABQ (0-24)	0.18**	0.10

Pearson's correlation coefficient; ***p ≤ 0.001, **0.001<p ≤ 0.01 *0.01<p ≤ 0.05

VAS, Visual Analogue Scale; BMI, Body Mass Index; P4 test, Posterior Pelvic Pain Provocation test; ASLR test, Active Straight Leg Raise test; HSCL-25, Hopkins Symptom Check List; DRI, Disability Rating Index; LBP, Low Back Pain; mFABQ, modified Fear Avoidance Beliefs Questionnaire

Pre-pregnancy BMI, smoking, physical activity before pregnancy, full time work and Beighton score for hypermobility were not significantly associated with DRI in gestation week 30 in the bivariate analysis (0.16 ≤ p ≤ 0.64). Physical activity before pregnancy, full time work and Beighton score for hypermobility were not significantly associated with pain intensity in gestation week 30 in the bivariate analysis (0.38 ≤ p ≤ 0.98). These variables were not entered in to the respective multivariable models. Age, gestation week, pre-pregnancy LBP, and work condition were not significantly associated with the response variables (0.11 ≤ p ≤ 0.65), but were entered into the multivariate models based on associations reported in previous studies [4-9].

In the multivariable model, pain locations, P4 test, sum of pain provocation tests, and HSCL-25 in early pregnancy were significantly associated with DRI in gestation week 30 (Table 4). Age, parity, marital status, education, use of contraceptive pills, the ASLR test, pre-pregnancy history of LBP, work condition, number of pain sites and mFABQ in early pregnancy were not significantly associated with DRI in gestation week 30 in the multivariable analyses (0.08 ≤ p ≤ 0.98). No significant interactions between the explanatory variables were found (0.21 ≤ p_interaction _≤ 0.97). When we adjusted for DRI in early pregnancy, R^2 ^increased from 0.26 (model 1) to 0.37 (model 2) and the sum of pain provocation tests and HSCL-25 were no longer significant (p = 0.26 and p = 0.49, respectively) (Table 4). Additional adjustment for gestation week at inclusion did not change the results.

**Table 4:** Associations between disability in gestation week 30 and risk factors measured in early pregnancy (n = 268).

	Crude estimates		Adjusted estimates; model 1		Adjusted estimates; model 2	
	β^1 ^(95% CI^2^)	p-value	β^1 ^(95% CI^2^)	p-value	β^1 ^(95% CI^2^)	p-value
***Pain locations***						
						
**No pain**	Reference	<0.001	Reference	0.007	Reference	0.03
**Symphysis pain only**	17.7 (6.8, 28.6)		14.0 (3.7, 24.1)		11.8 (2.3, 21.2)	
**Posterior pain only**	10.7 (6.2, 15.3)		4.8 (-0.2, 9.6)		3.4 (-1.0, 7.8)	
**Combined symphysis pain and posterior pain**	24.5 (15.6, 33.5)		11.8 (2.6, 21.0)		8.4 (-0.07, 17.0)	
***P4 test***						
						
**Negative**	Reference	<0.001	Reference	<0.001	Reference	<0.002
**Unilateral positive**	8.0 (2.6, 13.5)		2.2 (-3.4, 7.9)		3.3 (-1.9, 8.6)	
**Bilateral positive**	19.8 (14.3, 25.2)		12.0 (6.0, 18.0)		10.0 (4.4, 15.6)	
						
**Pain provocation tests (sum)**	5.3 (3.9, 6.7)	<0.001	1.7 (0.3, 3.0)	0.02	0.7 (-0.5, 2.0)	0.26
***HSCL-25***						
**<1.75**	Reference	<0.001	Reference	0.006	Reference	0.49
≥ **1.75**	14.0 (7.6, 20.3)		8.2 (2.3, 14.0)		2.0 (-3.7, 7.7)	
**DRI in early pregnancy**	0.6 (0.5, 0.7)	<0.001	-	-	0.5 (0.3, 0.6)	<0.001

^1^Estimated regression coefficients, ^2 ^CI, confidence interval. DRI, Disability Rating Index; P4 test, Posterior Pain Provocation test; HSCL-25, Hopkins Symptom Check List

In the multivariable model for pain intensity in gestation week 30 similar results were found (table 5). The same variables were significant except for HSCL-25. Age, parity, marital status, education, use of contraceptive pills, pre-pregnancy BMI, smoking, the ASLR test, pre-pregnancy history of LBP, work condition, number of pain sites, and mFABQ in early pregnancy were not associated with pain intensity in gestation week 30 in the multivariable analysis (0.07 ≤ p ≤ 0.80). No significant interactions between the explanatory variables were found (0.25 ≤ p_interaction _≤ 0.77). Adjustment for pain intensity in early pregnancy increased the R^2 ^from 0.29 (model 1) to 0.33 (model 2) and the sum of pain provocation tests in early pregnancy was no longer significant (p = 0.23) (table 5). Additional adjustment for gestation week in early pregnancy did not change the results.

**Table 5:** Associations between pain intensity (worst evening pain) gestation week 30 and risk factors measured in early pregnancy (n = 268).

	Crude estimates		Adjusted estimates; model 1		Adjusted estimates; model 2	
	β^1 ^(95% CI^2^)	p-value	β^1 ^(95% CI^2^)	p-value	β^1 ^(95% CI^2^)	p-value
***Pain locations***						
						
**No pain**	Reference	<0.001	Reference	<0.001	Reference	<0.001
**Symphysis pain only**	44.2 (27.7, 60.6)		40.4 (24.4, 56.5)		35.5 (19.7, 51.1)	
**Posterior pain only**	23.5 (16.6, 30.3)		15.3 (7.8, 22.8)		11.8 (4.3, 19.2)	
**Combined symphysis pain and posterior pain**	40.5 (26.9, 54.0)		26.0 (11.6, 40.4)		16.5 (1.8, 31.1)	
***P4 test***						
						
**Negative**	Reference	<0.001	Reference	0.07	Reference	0.01
**Unilateral positive**	16.5 (7.7, 25.2)		5.8 (-3.1, 14.8)		6.1 (-2.6, 14.7)	
**Bilateral positive**	28.6 (19.9, 37.3)		15.2 (5.8, 24.6)		13.7 (4.5, 22.8)	
**Pain provocation tests (sum)**	6.3 (4.5, 8.0)	<0.001	2.3 (0.3, 4.4)	0.03	1.3 (-0.8, 3.3)	0.23
**Pain intensity in early pregnancy (worst evening pain)**	0.7 (0.5, 0.8)	<0.001	-	-	0.4 (0.2, 0.5)	<0.001

^1^Estimated regression coefficients, ^2 ^CI, confidence intervals. P4, Posterior Pelvic Pain Provocation test

The effect estimates of each response variable were relatively large in both models, although the 95% confidence intervals were wide. Yet the effect estimates seemed to be higher for pain intensity compared with DRI. For instance our data shows that pain intensity in late pregnancy is 40.4 (95% CI: 24.4, 56.6) higher when pain was present in the symphysis only, compared with having no pain in early pregnancy and adjusted for P4 test and sum of pain provocation tests (table 5, model 1).

## Discussion

The main results from this study were that pain locations in the pelvis, positive P4 test and sum of pain provocation tests in early pregnancy were significantly associated with disability and pain intensity in late pregnancy. In addition, distress was significantly associated with disability. The functional test ASLR, fear avoidance beliefs and the number of pain sites were not significantly associated with neither disability nor pain intensity.

The risk factors identified in this study differ from those that have been reported before. Strenuous work, pre-pregnancy history of LBP and parity have previously been identified as risk factors for PGP in studies applying bivariate statistics [4-6] and multivariable models [9]. In our bivariate correlation analyses, the first two variables were not significantly associated to neither disability nor pain intensity in gestation week 30, while parity was. None of the variables were significant in the multivariable analyses. This could be due to difference in design or to the use of different levels of statistical methods. One possible explanation for the difference could be that previous studies have often recorded the risk factors retrospectively, late in pregnancy and after the onset of symptoms. Hence, the women's reporting of these factors might be biased by pain [7,8]. The prospective design of the present study ensured that this possible bias was avoided. At the time of inclusion and measurement of the risk factors, none of the women had defined their symptoms as a problem and they were not seeking treatment.

It is also noteworthy that the results of the functional ASLR test measured in early pregnancy, used with a distinction between those with strong affliction and those with none or lesser affliction, was not significantly associated with disability. This might indicate that severe impairment of motor control and movement of the legs relative to the pelvis was not important for the development of PGP. On the other hand, the response to the P4 test was identified as a risk factor for both pain intensity and disability. Since this test is supposed to elicit a distinct located pain deep in the gluteal area [17], it seems that affliction in the posterior pelvis has an impact on the course. This is, however, partly contradicted by the data from pain locations. Self-reported pain only in the symphysis in early pregnancy had about the same impact on disability and pain intensity in gestation week 30 as did combined symphysis pain and posterior pain. Moreover posterior pain (without symphysis pain) in early pregnancy was not significantly associated with disability and pain intensity in gestation week 30. Since this group was the largest, the lack of effect can hardly be explained by lower test power than the other pain locations. Although the confidence intervals were wide, our data indicate that subclinical afflictions in both anterior and posterior part of the pelvis are of importance for development of pain and disability. Hence, our data suggest that symphysis pain can be an early indicator or precursor for pain development in other areas of the pelvis. Interestingly, the association seems to disappear when pain location and disability are measured simultaneously in late pregnancy [3].

When we included disability or pain intensity assessed in early pregnancy in the multivariable models, some explanatory variables were no longer significant. This means that these variables were not risk factors for the change in disability or pain intensity. However, from a clinical point of view it is more important to identify risk factors for disability and pain intensity late in pregnancy than the change from early pregnancy. This is supported by the data showing an increased DRI already in early pregnancy compared with healthy non-pregnant women [20].

Several of the previously identified risk factors for PGP in pregnancy are similar to those reported for LBP and for other musculoskeletal disorders and are not specific for PGP [36,37]. These comprise socio-demographical factors, previous history of LBP, strenuous work and high level of distress. In contrast, positive response to the P4 test has been shown to be sensitive and specific for PGP [17]. Also the pattern of pain locations within the pelvis is probably specific for PGP, and one might therefore hypothesize that both the P4 test and pain locations are "condition specific" risk factors for PGP.

The response variables used in this study were measured as scale values whereas previous studies have used dichotomous responses for example reporting PGP or not. From the large variation in responses shown when using scales in the present study, one might question to what extent the dichotomous response variables actually reflects important affliction. The dichotomous response variables have resulted in very high prevalence rates for PGP in pregnancy [1,2,10,25]. We have recently found that the variability in DRI was large both for women reporting and not reporting PGP [3]. In order to capture associations to this large range of affliction, the used scales seem to provide additional information than the dichotomous responses.

Previous studies have shown associations between distress, fear avoidance beliefs and activity limitations in patients with LBP [38-42], and also that distress contributed to physical activity and work loss in an acute sample of LBP patients [36]. Our results showed that distress contributed into the model for disability but not for pain intensity. Interestingly the effect of HSCL-25 on disability in gestation week 30 disappeared when we controlled for disability at inclusion. As in the study from Grotle and co-workers of acute LBP [43], fear avoidance beliefs was not identified as a risk factor for either disability or pain intensity.

Over the years, there has been a growing evidence for predictive effect of widespread pain on long term changes in work disability [44]. Furthermore, it has also been reported that the risk of long-term work disability was lower for persons with localized LBP compared with persons with LBP combined with pain in other bodily areas. The risk for long-term work disability increased with the latter [45,46]. We included number of pain sites (excluding low back and pelvic area) in the multivariable analyses, and found that it did not contribute in any of the models. The lack of effects may be due to the small number of possible pain sites. However, it is also possible that PGP in pregnancy is a specific condition characterized by a rather short course compared with other musculoskeletal pain conditions. Most of the women recover shortly after delivery. One might thus speculate that multiple pain sites are not of importance for development of PGP in pregnancy, but could still be of importance for non-recovery from PGP postpartum.

The present study has several strengths, including the use of a prospective design, continuous response variables and multivariable statistics. Furthermore the implementation of clinical risk factors, use of blinded examiners and the follow-up of all pregnant women in the cohort independent of having PGP or not also strengthen the study.

A limitation that should be considered when interpreting the results is the limited numbers of women in some of the groups. However, even though the confidence intervals are wide, the findings indicate that the risk factors are of importance. On the other hand, lack of significant results should be interpreted with caution.

Another possible weakness could be the representativeness. The women participating in the cohort were about the same age and in the same gestation week as women declining participation. Women who were excluded from analyses due to late inclusion were also about the same age. The average age of women giving birth in Norway have been 30.3 years (2006 - 2007) [47] i.e., almost similar as in our cohort. There were some differences in the percentage of nulliparous women in the non-participant group, the excluded group and the participants (44%, 77% and 59% respectively). The number of nulliparous women in the cohort was also slightly higher than among Norwegian women (59% vs 42%). We cannot exclude the possibility that another cohort of pregnant women in Norway, would result in somewhat different results with regard to prevalence of pain locations and positive clinical tests. However, the associations between them are expected to be similar.

### Implications

Even though most women recover from PGP shortly after delivery, it has been shown that a number of women report pain for longer time periods and that some of them have serious problems [48-50]. Hence it seems important to identify risk factors for development of PGP in pregnancy that could contribute to better management and thereby prevent persistent disability after delivery. Risk factors identified in previous studies, such as parity and strenuous work can hardly be treated or managed for prevention purposes. The identification of the clinical risk factors in the present study therefore opens up new possibilities for management. Prevention and treatment of PGP in pregnancy would have considerable implications for the women, but also for the society in terms of productivity and health costs. However, it remains to be seen whether the risk factors identified in the present study are of clinical value in treatment and prevention of PGP.

## Conclusions

In conclusion, we have found that pain locations in the pelvis, bilateral positive P4 test, and sum of pain provocation tests in early pregnancy were significantly associated with disability and pain intensity in gestation week 30. The effect estimates were relatively large. Furthermore distress was significantly associated with disability, but not with pain intensity. Fear avoidance beliefs were not significantly associated with any of the responses. These results thus suggest that a clinical examination including a few tests performed in early pregnancy may identify women at risk of a more severe PGP late in pregnancy. The identification of clinical risk factors may provide a foundation for development of targeted prevention strategies.

## Competing interests

The authors declare that they have no competing interests.

## Authors' contributions

All authors contributed to the conception and design of the study. HSR, NKV and AMM obtained funding. HSR, NKV and MBV did the data analyses. All authors contributed to the interpretation of the results and critical revision of the manuscript for important intellectual content and approved the final version of the manuscript.

## Authors' informations

HSR (RPT and MSC) is doctoral student and manual therapist, MBV (PhD) is associated professor in biostatistics, AMM (RPT and PhD) is professor, NKV (PhD) is professor and Head of institute

## Pre-publication history

The pre-publication history for this paper can be accessed here:

http://www.biomedcentral.com/1471-2474/11/91/prepub
